# Zebrafish aversive taste co-receptor is expressed in both chemo- and mechanosensory cells and plays a role in lateral line development

**DOI:** 10.1038/s41598-017-14042-3

**Published:** 2017-10-18

**Authors:** Nazia Mojib, Jin Xu, Zinka Bartolek, Barry Imhoff, Nael A. McCarty, Chong Hyun Shin, Julia Kubanek

**Affiliations:** 1 School of Biological Sciences, Georgia Institute of Technology, Atlanta, GA 30332 USA; 2 Wallace H. Coulter Department of Biomedical Engineering, Georgia Institute of Technology, Atlanta, GA 30332 USA; 3 Department of Pediatrics, Emory University and Children’s Healthcare of Atlanta Center for Cystic Fibrosis and Airways Disease Research, Atlanta, GA 30322 USA; 4 Parker H. Petit Institute for Bioengineering and Bioscience, Georgia Institute of Technology, Atlanta, GA 30332 USA; 5 Aquatic Chemical Ecology Center, Georgia Institute of Technology, Atlanta, GA 30332 USA; 6 School of Chemistry and Biochemistry, Georgia Institute of Technology, Atlanta, GA 30332 USA

## Abstract

Fishes rely on both chemical and tactile senses to orient themselves to avoid predators, and to detect and taste food. This is likely achieved by highly coordinated reception of signals by mechano- and chemosensory receptors in fish. A small co-receptor from zebrafish, receptor activity modifying protein (RAMP)-like triterpene glycoside receptor (RL-TGR), was previously found to be involved in recognition of triterpene glycosides, a family of naturally occurring compounds that act as chemical defenses in various prey species. However, its localization, function, and how it impacts sensory organ development *in vivo* is not known. Here we show that RL-TGR is expressed in zebrafish in both i) apical microvilli of the chemosensory cells of taste buds including the epithelium of lips and olfactory epithelium, and ii) mechanosensory cells of neuromasts belonging to the lateral line system. Loss-of-function analyses of RL-TGR resulted in significantly decreased number of neuromasts in the posterior lateral line system and decreased body length, suggesting that RL-TGR is involved in deposition and migration of the neuromasts. Collectively, these results provide the first *in vivo* genetic evidence of sensory cell-specific expression of this unusual co-receptor and reveal its additional role in the lateral line development in zebrafish.

## Introduction

Animals use diverse sensory systems for mediating critical ecological relationships, for example between predators and their prey (food)^1^. These systems help animals locate food, gauge its nutritional value, and avoid noxious items. However, the molecular machinery behind the signal reception mediated by these systems is relatively understudied. In fishes, a critical behavior is rejection of prey-containing noxious chemicals, a process known as aversive chemoreception. One challenge with aversive chemoreception is that toxic and deterrent compounds are structurally diverse, likely more so than the common taste molecules that cue fishes to palatable foods^2^. Plant and animal prey of fishes are commonly defended by species-specific molecules representing diverse structural classes, including peptides, alkaloids, polyketides, isoprenoids, and hybrids of these^3^. Therefore, an aversive chemoreception system needs to be able to identify a wide range of ligand structures.

An efficient and versatile way for a chemosensory system to recognize numerous, structurally distinct molecules would be through a combinatorial system in which a small number of receptors and co-receptors couple in various ways to enable detection of diverse noxious molecules belonging to different structural classes. Indeed, fish genomes appear to encode fewer chemoreceptors than one might expect when compared with other vertebrates^4^. One such system of chemoreception involving receptor and co-receptor was recently discovered in zebrafish. Through heterologous expression in *Xenopus* oocytes, a small, transmembrane co-receptor from zebrafish was found to be involved in the recognition of a family of compounds that act as feeding deterrents^5^. RAMP-like triterpene glycoside receptor (RL-TGR) is a 96 amino acid protein that, when complexed with an unidentified zebrafish G-protein coupled receptor (GPCR), transduces a signal in the presence of triterpene glycoside ligands. The location and distribution of RL-TGR are important factors in understanding the role of this receptor in organism-environment interactions, and are expected to affect the kinds of ligand(s) RL-TGR can recognize and the message being transduced. The ligands could be cues from the external environment as is the case for chemical compounds in food or they could be secreted by cells as in the case of interleukins, growth factors, etc.^5,6^.

Given its proposed function in chemoreception of prey chemical defenses, RL-TGR expression is expected to be localized in chemosensory tissues, namely olfactory epithelium and taste buds in the mouth. The taste buds allow fish to discriminate between palatable and noxious food items, and are found throughout the oropharyngeal epithelium, on the branchial arches, and on the head and other regions of the body in some species^7^. In this study, we sought to first determine the location and distribution pattern of RL-TGR in zebrafish in order to dissect the molecular basis of its cellular function in *vivo*. Further, we performed morpholino-mediated knockdown of RL-TGR to reveal its physiological effects on early development of zebrafish.

## Results

### Expression pattern of RL-TGR in zebrafish

**Figure 1:**
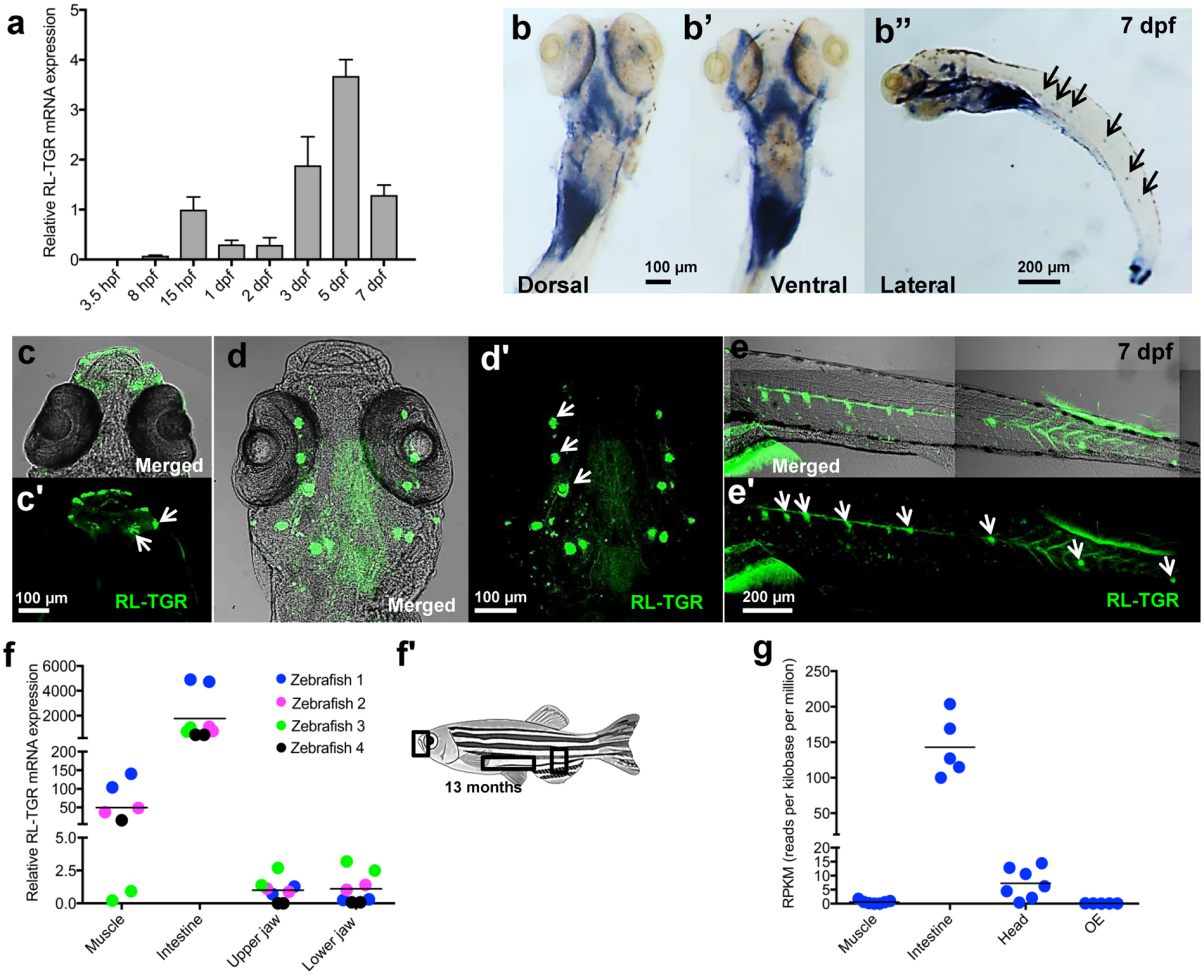
Expression of RL-TGR in zebrafish. (**a**) RT-qPCR analysis of *rltgr* in the early developmental stages of zebrafish, 3.5 hours post fertilization (hpf) to 7 days post fertilization (dpf). Gene expression was normalized to that of 18 S rRNA and presented as fold changes (mean ± SD) against 15 hpf expression. *rltgr* transcripts, which were detected by RT-qPCR analysis of whole embryos, begin to express from 8 hpf stage onwards. (**b**-**b”**) Whole mount *in situ* hybridization (WISH) showing the expression of *rltgr* at 7 dpf (dorsal, ventral, and lateral view, respectively). At 7 dpf, *rltgr* is expressed in both lateral as well as posterior part of pharyngeal region and intestine (**b**-**b’**, dorsal and ventral view, anterior to the top). Additionally, *rltgr* is expressed in the neuromasts of the lateral line system (black arrows, b”, lateral view, anterior to the left). (**c**–**e’**) Immunostaining of RL-TGR at 7 dpf in wild type embryos. RL-TGR-positive cells were detected (**c**-**c’**, dorsal view, anterior to the top) in the lips and neuromasts (white arrows) of the mouth region, (**d**-**d’**, ventral view, anterior to the top) neuromasts (white arrows) of the cephalic lateral line, and (**e**-**e’**, lateral view, anterior to the left) neuromasts (white arrows) of the posterior lateral line system. (**f**) Tissue specific expression of *rltgr* in adult zebrafish by RT-qPCR. Gene expression was normalized to that of 18 S rRNA and presented as fold changes (mean ± SD) against expression in muscle (4 biological replicates). The horizontal lines show median values. Corresponding to WISH results, the highest expression of *rltgr* was observed in the intestine followed by expression in muscles and mouth (upper and lower jaw). (**f’**) Image of adult zebrafish marking tissues analyzed for RT-qPCR in (**f**), adapted from Wikipedia commons^32^ with the license https://creativecommons.org/licenses/by/3.0/deed.en) (changed to grayscale). (**g**) Expression of *rltgr* in RNA Sequencing data of different tissues from independent studies available in short read archive database (SRA-NCBI). Read counts were normalized across the libraries for comparison. *rltgr* expression was observed in intestine, muscle, head and olfactory epithelium (OE). The different tissues of adult zebrafish harvested for total RNA extraction are depicted as a schematic diagram. (**c**–**e’**) are confocal single-plane images. n > 20 embryos; scale bars are shown in individual images.

Reverse transcriptase quantitative PCR (RT-qPCR) analysis in zebrafish whole embryos showed that *rltgr* starts to be expressed at 8 hours post fertilization (hpf) with a major increase at 3 days post fertilization (dpf) onwards (Fig. 1a). Using whole-mount *in situ* hybridization (WISH) labeling, spatiotemporal *rltgr* expression was detected from 8 hpf to 19 dpf (Fig. S1). Predominantly, *rltgr* expression was detected in the head, pharyngeal region, and intestine (7 dpf embryo) (Fig. 1b,b’). Its expression was also detected in the neuromasts of the posterior lateral line system (Fig. 1b”). From 7 dpf onwards, *rltgr* was detected by WISH in the pharyngeal region, intestine, and neuromasts, a pattern observed until at least 19 dpf (Fig. S1).

To analyze the expression of RL-TGR protein in zebrafish embryos, we performed whole-mount immunofluorescence experiments. To this end, we generated a rabbit polyclonal antibody against a peptide containing the 18 amino acid residues (KYEDALKVTVLKFVKHLE) from the C-terminal portion of the predicted transmembrane domain of RL-TGR, which showed immunoreactivity with the recombinant RL-TGR protein expressed in HEK 293 cells (Fig. S2). We further evaluated the specificity of the anti-RL-TGR antibody by immunostaining the cichlid (*Maylandia zebra*) embryos with anti-RL-TGR antibody (Fig. S3). Because the *M. zebra* genome (GenBank assembly accession: GCA_000238955) sequence does not contain the *rltgr* gene, it serves as an ideal negative control to evaluate the specificity of our custom generated anti-RL-TGR antibody. As expected, there was no immunoreactivity in cichlid embryos at both 3 and 7 dpf with anti-RL-TGR antibody confirming its specificity with RL-TGR protein (Fig. S3). However, we were unable to obtain specific reactivity via western blot using this antibody.

In zebrafish, RL-TGR-positive cells were detected in the epithelium of the upper and lower lips (Fig. 1c,c’), as well as in neuromasts of both the cephalic (Fig. 1d,d’) and posterior (Fig. 1e,e’) lateral line system, as evidenced by immunostaining of the 7 dpf embryos with anti-RL-TGR antibody. Consistent with the gene expression findings, RL-TGR-positive cells were detected throughout the embryos at 3–18 hpf (Fig. S4a–c). In 1 dpf embryos, RL-TGR-positive cells were detected in the nasal sensory epithelium of the olfactory pit (Fig. S4d). In 2 dpf embryos, immunofluorescence was detected both in the olfactory pit and in the brain (Fig. S4e,f). In 3 to 7 dpf embryos, RL-TGR immunostaining signal occurred in the epithelium of upper and lower lips, olfactory pit, and neuromasts (Fig. S4g–l). The number and size of anti-RL-TGR-labeled neuromasts in the anterior and posterior lateral line were increased in 7 dpf larvae (Fig. S4k,l). These RL-TGR protein expression patterns are in agreement with the *rltgr* gene expression patterns in different sensory tissues (Fig. 1b–b”) and point to a function of RL-TGR protein in the zebrafish sensory system during early development.

In adult zebrafish, tissue-specific expression of the *rltgr* gene evaluated using RT-qPCR corroborated observations with 5-19 dpf stage larvae (Fig. S1), whereby *rltgr* is expressed in the intestine and mouth (both upper and lower jaw including lips) (Fig. 1f,f’). In adults, *rltgr* expression was also detected in muscle which could be due to the presence of neuromasts on the body surface. We validated our expression results by analyzing the expression of *rltgr* in different tissue-specific zebrafish transcriptomes from publicly available RNASeq data (Fig. 1g). Consistent with the tissue-specific expression, *rltgr* is highly expressed in the intestine and head. However, insignificant numbers of reads were detected via this approach from the olfactory epithelium (OE) and muscle (Fig. 1g).

### RL-TGR is expressed in zebrafish taste buds, lips, and olfactory epithelium

**Figure 2:**
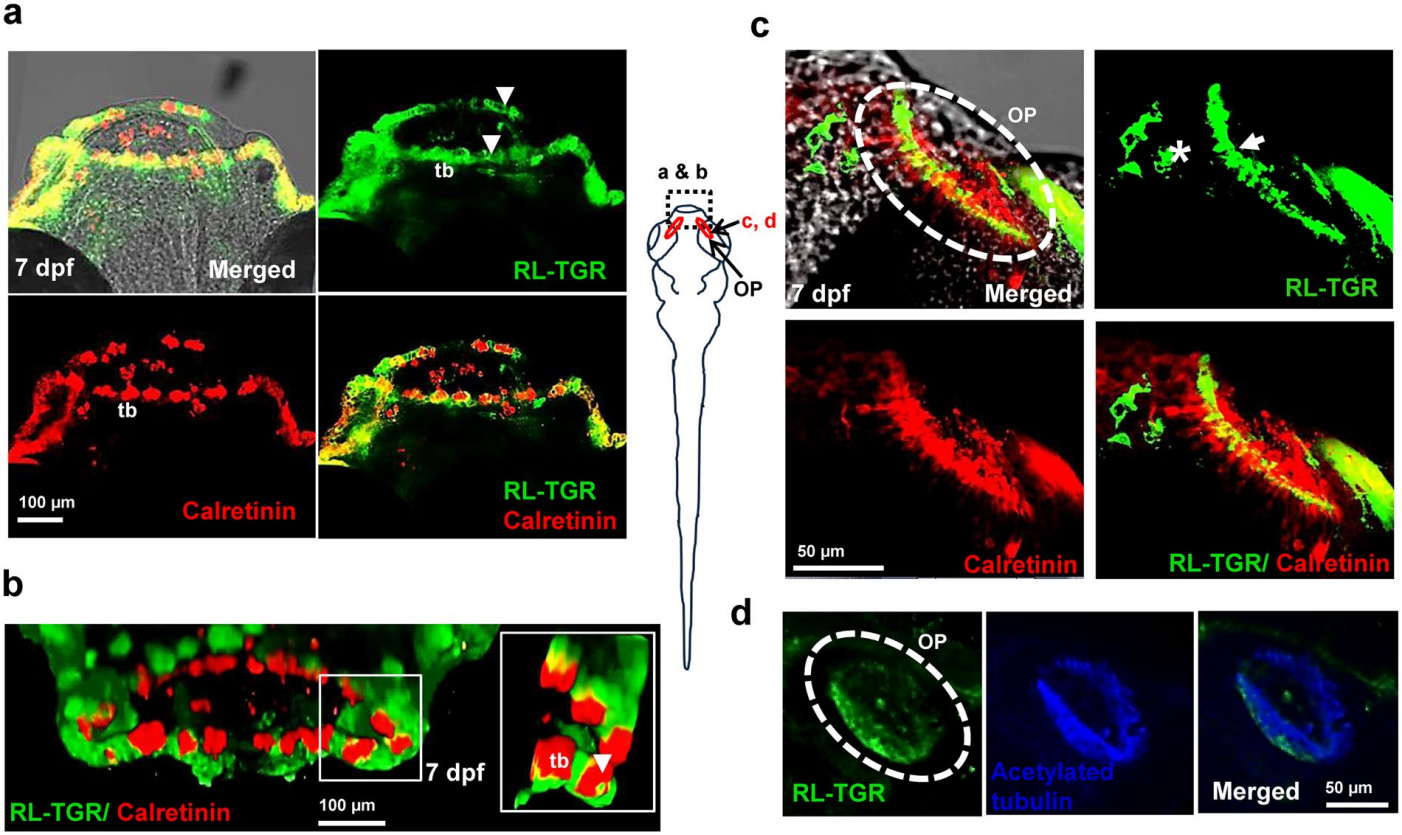
RL-TGR is expressed in the lip epithelium, taste buds, and olfactory epithelium. (**a**) Confocal images of the mouths of wild type embryos (dorsal view, anterior to the top) at 7 dpf stained with RL-TGR (green; expression in the epithelial layer of both upper and lower lip indicated by white arrowheads, basal cells and apical microvilli of the taste buds marked as ‘tb’) and taste bud marker calretinin (red, expression in labial taste buds, also marked as ‘tb’). (**b**) 3-D confocal images of the lips of 7 dpf wild type embryos (anterior of the lip facing outward) stained with RL-TGR (green) and calretinin (red). RL-TGR-positive cells were detected in the lip epithelium, basal cells, and apical microvilli (white arrowheads in the zoomed in inset) of taste buds (tb). (**c**) Confocal images of olfactory pit (OP) of wild type embryos (dorsal view, anterior to the top) stained with RL-TGR (green; expression in the sensory epithelium of the olfactory pit outlined by white dotted circles) and calretinin (red, expression in the olfactory receptor neurons of the olfactory epithelium). (**d**) Confocal images of olfactory pit (OP) of wild type embryos (dorsal view, anterior to the top) stained with RL-TGR (green; expression in the sensory epithelium of the olfactory pit outlined by white dotted circles) and acetylated tubulin (blue, expression in the cilia of the olfactory epithelium). n > 20 embryos; schematic diagram of the 7 dpf larvae shows the location at 7 dpf of different images; the scale bars are shown in individual images.

The taste buds with open receptor areas appear in the larval zebrafish mouth and oropharyngeal cavity around 4-5 dpf^7^, when they start feeding. Therefore, we examined the upper and lower lips in 7 dpf embryos and used calretinin, a calcium binding protein, as a taste bud marker. RL-TGR-positive cells were localized in the epithelial layer of both upper and lower lips (Fig. 2a) and colocalized with calretinin in the basal cells and microvilli (tip or receptor area) of the taste buds in the upper and lower lips (Fig. 2b). RL-TGR-positive cells were also located in the nasal sensory epithelium of the olfactory pit (Fig. 2c) and colocalized with calretinin in the sensory epithelium region where both are expressed in the olfactory receptor neurons of the olfactory pit. Using acetylated tubulin as a marker for ciliated cells, we found that RL-TGR-positive cells were located in the ciliated cells of the olfactory epithelium (Fig. 2d).

### RL-TGR is expressed in zebrafish mechanosensory neuromasts

**Figure 3:**
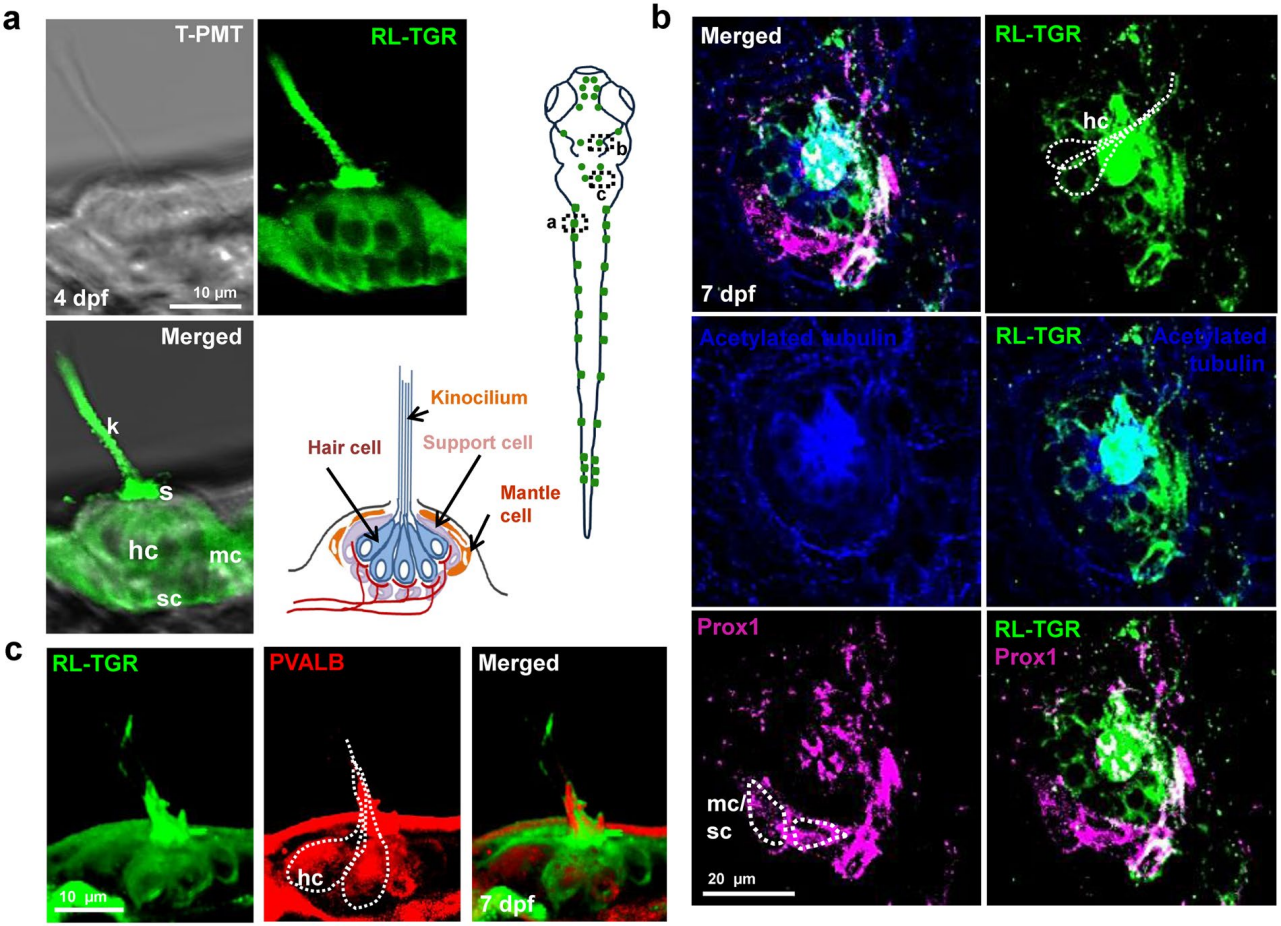
RL-TGR is expressed in the neuromasts. (**a**) Confocal images of a single neuromast of the posterior lateral line system of wild type embryo at 4 dpf (lateral view). RL-TGR (green) staining signal was detected in hair cells (hc), support cells (sc), and mantle cells (mc) of the neuromast. The image on the bottom right represents the scheme of a neuromast illustrating different cells and their organization (modified after Ghysen and Dambly-Chaudiere, Current Opinion in Neurobiology, 2004)^33^. (**b**) Confocal images of a neuromast of wild type embryo (dorso-lateral view) at 7 dpf stained with RL-TGR (green, expression in the hair cells), acetylated tubulin (blue, expression in the cilia of the hair cells), and neuromast marker Prox1 (magenta, expression in the hair cells, supporting cells, and mantle cells). Colocalization of RL-TGR and acetylated tubulin suggests that RL-TGR is highly expressed in both kinocilia and cell bodies of the mechanosensory hair cells. RL-TGR colocalized with Prox1 in the stereocilia of the hair cells and supporting cells surrounding the neuromast. (**c**) 3D maximum intensity projection images of a neuromast of wild type embryo (lateral view) at 7 dpf stained with RL-TGR (green, expression in the hair cells), parvalbumin (PVALB) (red, expression in the hair cells). n > 50 embryos; schematic diagram of the 7 dpf larvae shows the location of different images; scale bars are shown in the images.

Neuromasts of the lateral line system sense hydrodynamic signals of water currents and help fish to orient, avoid predators, and detect food^8^. Immunostaining with RL-TGR antibody revealed that RL-TGR is expressed in neuromasts of the lateral line system. RL-TGR-positive cells were arranged in a typical rosette pattern, indicating expression of RL-TGR in hair cells, support cells, and mantle cells of the neuromast (Fig. 3a). Further, colocalization of RL-TGR and acetylated tubulin confirmed the localization of RL-TGR in both kinocilia and cell body of the hair cells (Fig. 3b). Additionally, colocalization of RL-TGR with Prospero-related homeobox gene 1 (Prox1), a transcription factor expressed in the lateral line primordium and used as a marker for neuromasts, confirmed the presence of RL-TGR in the stereocilia of the hair cells and in progenitor cells (mantle or supporting) surrounding neuromasts. The colocalized expression of RL-TGR and parvalbumin in the hair cells further confirmed the strong expression of RL-TGR in the hair cells of the neuromast (Fig. 3c). The expression of RL-TGR in the lateral line system suggests its potential signaling role in the development of neuromasts and/or their stereotypical distribution and function.

### Loss of RL-TGR activity affects lateral line development

**Figure 4:**
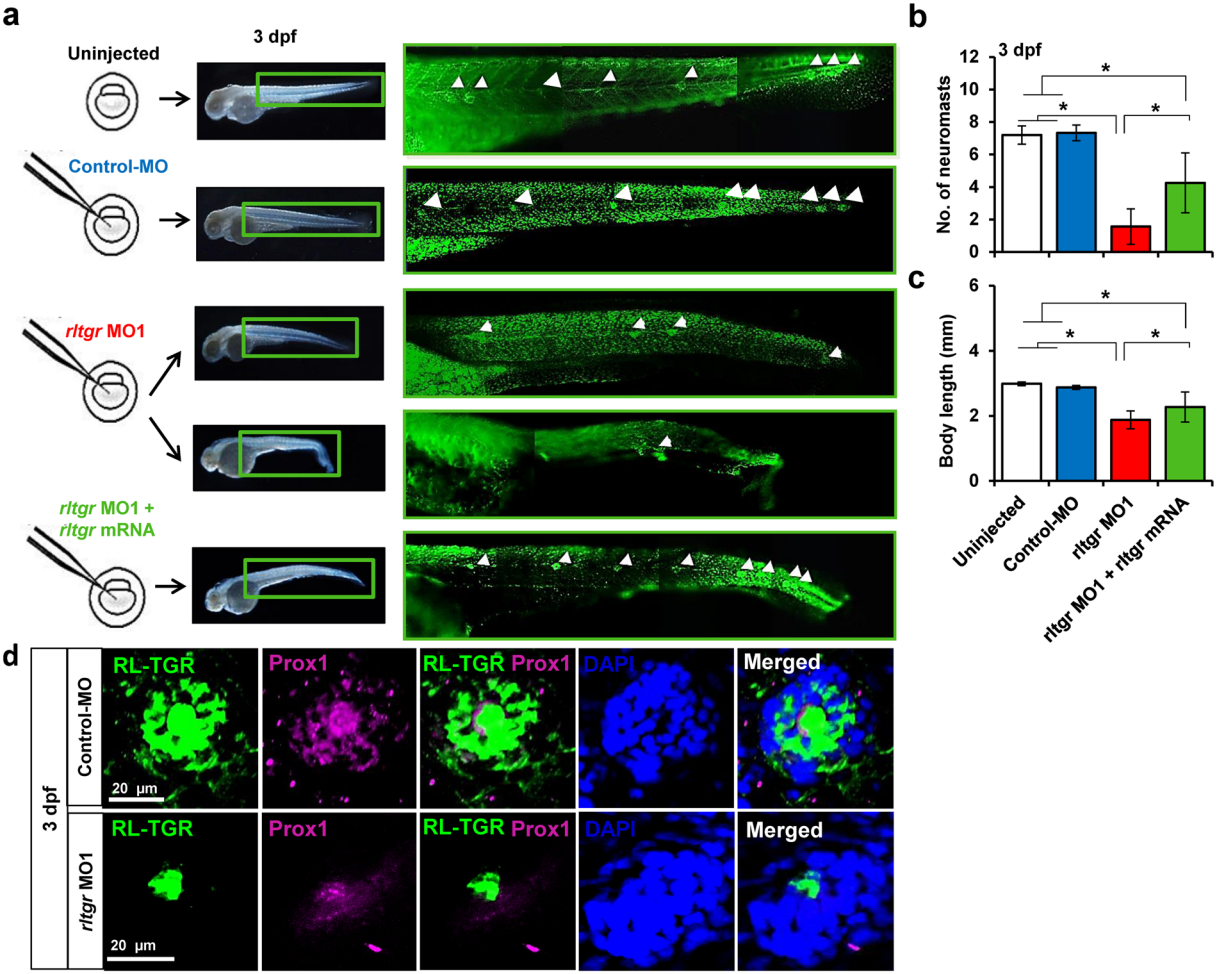
RL-TGR is involved in lateral line development. (**a**) Scheme showing injections of control MO, *rltgr* MO1 and *rltgr* mRNA in 1-2 cell stage embryos. At 3 dpf, the neuromasts were assessed (white arrowheads) in the respective injected populations by observing the embryos stained with SYTOX green nucleic acid stain. There was a decrease in the number of neuromasts in the *rltgr* morphants and the number was restored upon co-injection with *rltgr* mRNA (**b**). The bar graph shows that rescue in context with the number of neuromasts was significant (*P value < 0.0001; n = 15–16). (**c**) The body length of the injected populations is shown as a bar graph. Note a significant restoration of body length of *rltgr* morphants when co injected with *rltgr* mRNA (*P value < 0.0001; n = 35–66). (**d**) Confocal images of a single neuromast from 3 dpf control embryos and *rltgr* morphants (dorsal view) stained with RL-TGR (green) and neuromast marker Prox1 (magenta). DAPI stains nuclei (blue). n > 20; scale bars are shown in the images.

In order to investigate the role of RL-TGR in embryonic development, we performed knockdown experiments by injecting translation-blocking antisense *rltgr* morpholino oligonucleotides (*rltgr* MO1) in 1- to 2-cell stage embryos. We could not generate splicing-blocking morpholino oligonucleotides because *rltgr* is coded within a single exon. Compared to control MO-injected embryos (control embryos), *rltgr* MO1-injected embryos (morphants) exhibited a significant decrease in the immunostaining fluorescence intensity using the anti-RL-TGR antibody, demonstrating the MO’s protein translation blocking efficiency (Fig. S5). At both 3 and 5 dpf, control embryos did not exhibit any significant morphological defects (Fig. S6). In contrast, the morphants exhibited dose-dependent phenotypes characterized by a shortened anterior–posterior axis (body length), bent trunks, and curved tails (Fig. S6a). The observed *rltgr* MO1 dose-dependent phenotype revealed significant differences in the RL-TGR morphants compared to control-MO injected individuals at 3 and 5 dpf (P < 0.0001) (Fig. S6b). A significant decrease in the body length (at 3 dpf, P < 0.01; 5 dpf, P < 0.0001) and number of neuromasts (at 5 dpf, P < 0.0001) were observed in an *rltgr* MO1 dose-dependent manner (Fig. S7a,b). Injection of *rltgr* MO1 caused a decrease in the number of deposited neuromasts in the posterior lateral line, which was efficiently rescued by *rltgr* mRNA (Fig. 4a,b). Moreover, the body length was also restored upon co-injection with *rltgr* mRNA (Fig. 4a,c). It should be noted that the morpholino oligonucleotide (*rltgr* MO1) was designed to target the region upstream of start codon and that the mRNA used in the rescue experiment contained ORF of *rltgr* and not the morpholino target site, in order to ensure that there was no titration effect leading to false positive rescue results. It should also be noted that less severe phenotypes (short and curved body) were used for statistical purposes because it was difficult to assess the number of neuromasts in the more severe phenotypes with deformed posterior structures; this bias made the contrast more conservative. RL-TGR expression analysis in *rltgr* morphants revealed that RL-TGR-positive cells were confined to hair cells in the neuromasts (Fig. 4d). There was also a decrease in the expression of the neuromast marker Prox1 in *rltgr* morphants (Figs S5 and 4d).

### Loss of RL-TGR activity has little effect on taste bud development

**Figure 5:**
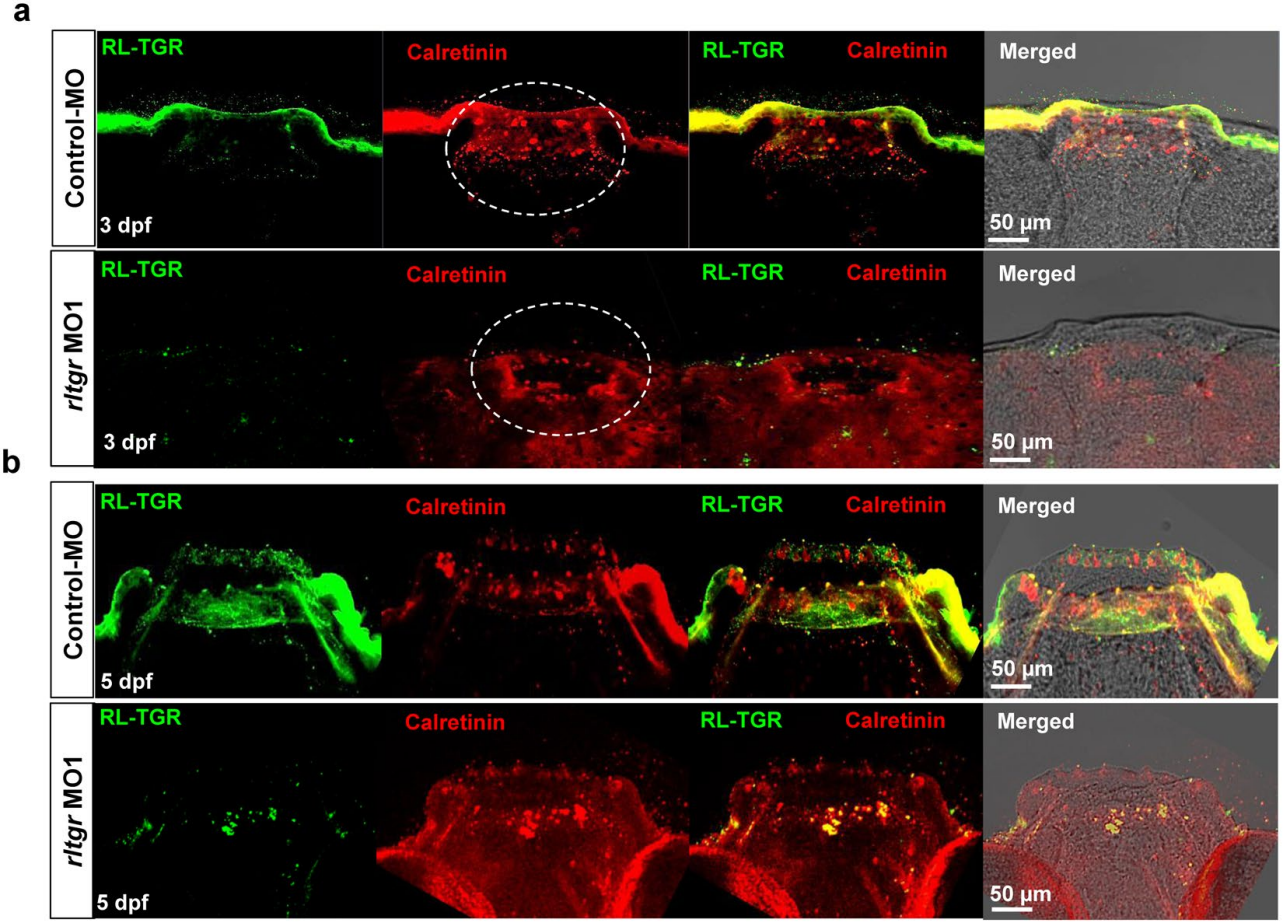
RL-TGR has a minimal effect on taste bud development. (**a**) Confocal images of the mouths of control embryos and *rltgr* morphants (dorsal view, anterior to the top) at 3 dpf stained with RL-TGR (green) and the taste bud marker calretinin (red). (**b**) Confocal images of mouth of control embryos and *rltgr* morphants (dorsal view, anterior to the top) at 5 dpf stained with RL-TGR (green) and taste bud marker calretinin (red). n > 10; scale bars are shown in the images.

Although RL-TGR activity is required for the lateral line development, it did not seem to affect the development of taste buds. At both 3 and 5 dpf, a majority of the morphants showed unaltered morphology of the mouth and structure of the taste buds (Fig. 5a,b), whereas a small population of morphants with severe body phenotypes exhibited deformity in the mouth with either no or underdeveloped taste buds. In both cases, the expression of RL-TGR was almost absent in the lip epithelium and tip of the taste buds, while the expression of calretinin was not affected in the lip epithelium and taste buds (Fig. 5).

### Validation of translation-blocking morpholino knockdown of *rltgr*

**Figure 6:**
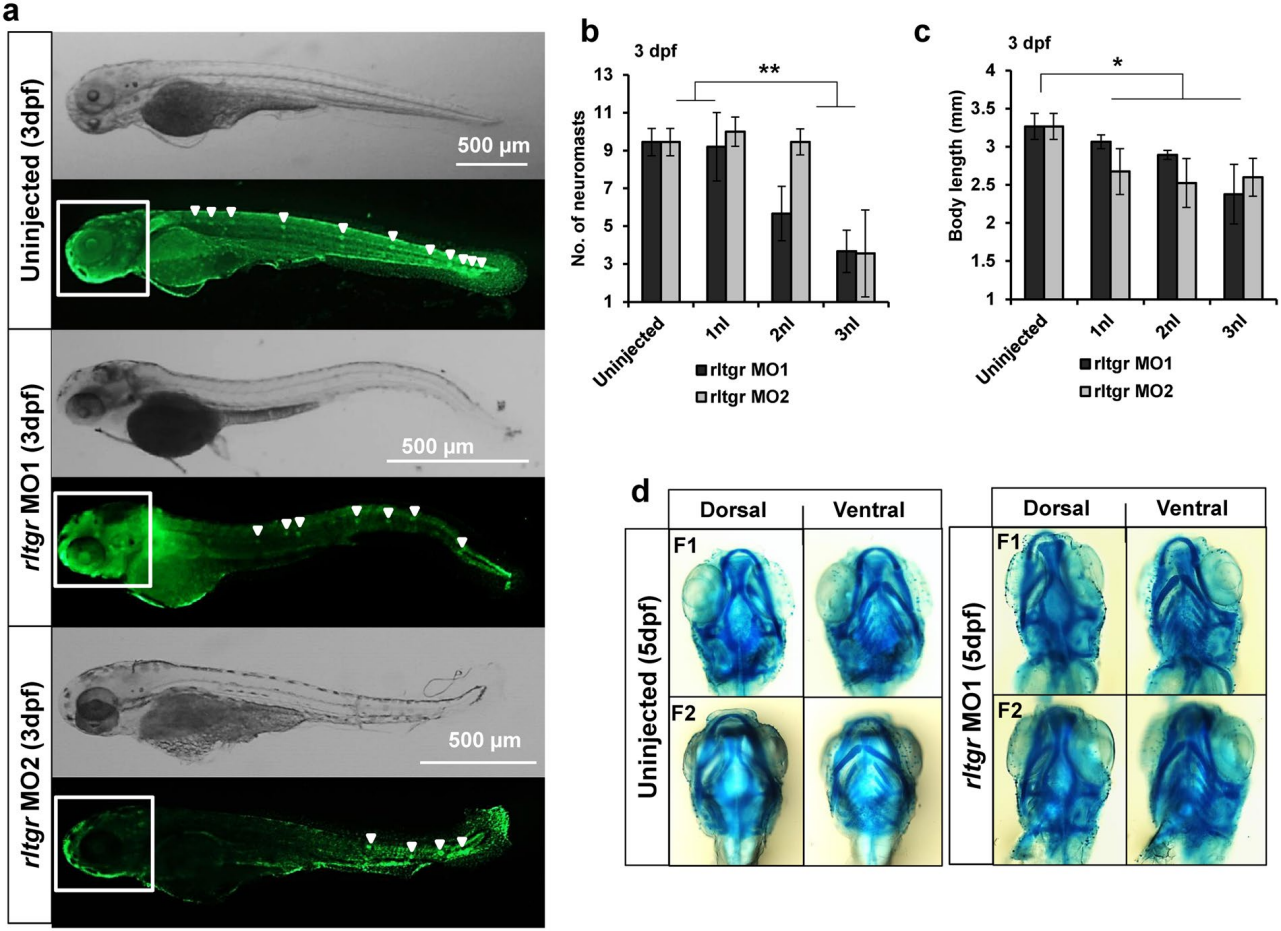
Demonstration of RL-TGR MOs specificity. (**a**) Brightfield and fluorescence representative images from uninjected, *rltgr* MO1, and *rltgr* MO2 injected embryos population at 3 dpf. The neuromasts were assessed (white arrowheads) in the respective injected populations by observing the embryos stained with SYTOX green nucleic acid stain. There was a decrease in the number of neuromasts in the *rltgr* morphants (both MO1 and MO2) as compared to uninjected embryo. (**b**) Quantitative assessment of the neuromasts shows that there was significant decrease in the number of neuromasts in both *rltgr* MO1 and MO2 injected populations (**P value <0.0001; n = 10–12). (**c**) Measurement of the body length shows that there was significant reduction in both *rltgr* MO1 and MO2 injected populations as compared to uninjected ones (*P value < 0.01; n = 26–30). (**d**) Brightfield images of 5dpf embryo heads stained with alcian blue cartilage stain to assess cartilage development (F1 = fish #1; F2 = fish #2). No delay in the cartilage development was observed in the morphants when compared to uninjected ones. n > 10; scale bars are shown in the images.

In order to confirm that the observed phenotypes of RL-TGR knockdown zebrafish juveniles were due to specific interaction between the morpholino (*rltgr* MO1) and the target sequence (*rltgr*), a second, non-overlapping, translation-blocking morpholino *rltgr* MO2 was designed and tested. We observed the same effect as with *rltgr* MO1 (Fig. 6). Both *rltgr* MO1 and MO2 morphants exhibited phenotypes characterized by a shortened anterior–posterior axis (body length), bent trunks, and curved tails with decreased number of lateral neuromasts (Fig. 6a). Yet, the head neuromasts developed normally in uninjected individuals as well as morphants (using either MO1 or MO2) suggesting that knockdown of *rltgr* affected the migration of posterior lateral line neuromast primordium only. At 3 dpf, a significant dose-dependent decrease in the body length and number of neuromasts was observed in embryos injected with *rltgr* MO1 or *rltgr* MO2 (Fig. 6b,c). Another potential side effect of morpholino treatment could be toxicity to cells which may have affected elongation of the body axis. To rule out the general development delay in morpholino-injected larvae arising from morpholino toxicity, we performed alcian blue cartilage staining at 5 dpf. We observed that cartilage development was not affected in both control and morpholino injected embryos (Fig. 6d). This suggests that the observed reduction in body size was not due to developmental delay.

## Discussion

### RL-TGR expression in both chemo- and mechanosensory cells

The observation that RL-TGR is expressed in chemosensory tissues *in vivo* such as lip epithelium, taste buds, and olfactory epithelium (Fig. 2) supports its role as a chemoreceptor which was previously indicated by heterologous expression in *Xenopus* oocytes^5^. Given that RL-TGR functions in conjunction with a GPCR^5^, it is unlikely that RL-TGR senses both chemical and tactile signals since mechanical stimuli may not be expected to be transduced via GPCRs due to their relatively slow signaling cascades. However, the lateral line system, typically implicated in mechanosensation, has also been reported to play a role in feeding, particularly in prey and food detection^9^. Even though each sensory system is sufficient by itself to accomplish behavioral objectives, fishes can modulate their responses using different sensory systems that might complement each other’s function to achieve complex behavior^10–12^. Feeding behavior in fishes is stimulated by integration of multiple types of cues providing information about prey suitability, prey location, and other aspects of the foraging environment, all dependent upon functioning visual, chemo- and mechanosensory systems^4,9,13^. Further, spatiotemporal expression profile of *rltgr* (WISH) (Fig. S1, 1b), qPCR and RNASeq data (Fig. f&g) revealed that it is also strongly expressed in the intestine. The expression of *rltgr* in zebrafish gut is consistent with multiple findings of taste receptors expressed in the guts of mammals and insects^14–16^. These receptors may function as a second line of defense against potentially noxious compounds as a part of enteric nervous system, supporting the hypothesis that RL-TGR functions in aversive taste reception.

### Possible roles of RL-TGR in the taste buds and olfactory epithelium

Identified previously as a co-receptor for sensing chemicals^5^, RL-TGR expression in the apical microvilli of taste buds protruding from the epithelium (open taste receptor area) (Fig. 2) justifies its role in sensing chemical compounds. In the apical microvilli of taste buds, its expression colocalizes with calretinin, a calcium binding protein that regulates intracellular Ca^2+^ homeostasis. Calretinin has been found to be widely distributed in the central and peripheral nervous system as well as in chemosensory cells such as taste buds and olfactory epithelium^17^. RL-TGR is expressed in the labial epithelial cells embedding the taste buds (Fig. 2). It was observed that most calretinin-positive cells do not express RL-TGR in the taste buds (Fig. 2b) which could be due to the difference in the origin of the cells. Calretinin-positive cells in the taste buds may have an endodermal origin^18^, whereas RL-TGR positive basal epithelial cells arise from the ectoderm^19^.

Since the cells of taste buds have a limited life span, they are maintained by continuous proliferation of epithelial progenitor and stem cells^20^. In mice, basal keratinocytes originating from the epithelial bilayer serve as progenitor populations that supply new cells to taste buds^21^. Even though the specific role of RL-TGR in the basal epithelial region of the taste buds is presently unclear, the expression pattern suggests that it may be involved in signaling in the progenitor or stem cell populations during taste bud development and/or regeneration. However, RL-TGR signaling may not be indispensable as the RL-TGR loss-of-function phenotype (less severe individuals with shortened bodies) had normally developed taste buds and lip epithelia (Fig. 5). Nevertheless, we did find mouth deformity with less or underdeveloped labial taste buds in morphants displaying the more severe phenotype (*data not shown*).

It has been reported that olfactory organs in fishes are formed much earlier in development than taste buds or gustatory organs^22^. Specifically, the receptors that bind odorants start to develop within 24 to 30 hpf (before hatching)^22^. RL-TGR co-receptor expression is consistent with the development pattern of chemosensory organs including both olfaction and taste. RL-TGR expression was first detected in olfactory epithelium at 1 dpf and then at 2 dpf (Fig. S4d,e). RL-TGR expression was found in the lip epithelium from 3 dpf onwards, consistent with prior observations that zebrafish taste buds develop starting at 3 dpf^7^. This order of onset of RL-TGR expression in parallel with different sensory modalities suggests one possibility that RL-TGR may help zebrafish larvae detect chemosensory cues via its expression in olfactory epithelium, and later helps larvae evaluate the quality of food through expression in taste buds when fish are actually able to feed.

### RL-TGR is involved in lateral line development

The function of a co-receptor is expected to depend on the kinds of ligand(s) it binds and the stimulus that is being transduced. The ligands can be from the external environment as is the case for chemical compounds in food or they can be ligands secreted by the cells such as interleukins and growth factors. In the latter case, co-receptors have been reported to be involved in chemical signaling during cell proliferation, migration, and embryonic development^6,23^. The zebrafish phenotype observed upon knockdown of RL-TGR, in which deposition of neuromasts was impaired (Fig. 4), points towards a likely role of RL-TGR in neuromasts. RL-TGR, a chemoreceptor, may sense a chemotactic gradient necessary for lateral line primordium migration. The primordium is a group of migrating epithelial cells that differentiates into neuromasts which requires coordination of diverse cellular behaviors via chemical signaling^24^. RL-TGR expression was observed in the primordium of the lateral line (*data not shown*). Recently, GPCR-mediated signaling was discovered in the context of lateral line development^25,26^, whereby G-proteins induce primordium migration through chemokine receptors, Cxcr4. Given that RL-TGR functions in conjunction with a yet-unidentified zebrafish GPCR^5^, RL-TGR may be participating in pathways controlled and regulated by GPCRs at different levels to promote migration of the lateral line primordium. Therefore, disruption in this activity by RL-TGR knockdown might have led to impaired migration and decreased number of deposited neuromasts (Figs 4 and 6). However, we cannot rule out the possibility that the lateral line phenotype and decreased body length could be indirect effects of RL-TGR knockdown impacting signaling factors, such as fibroblast growth factors or cysteine-rich glycoproteins (Wnt), important for lateral line development.

In a previous study, the role of RL-TGR in chemoreception was identified using a heterologous system, whereas the location, distribution, and function of RL-TGR in sensory organs *in vivo* are established for the first time in the current study. Herein we report that RL-TGR is expressed in sensory specific cells of both chemo- and mechanosensory organs. More importantly, a new role for RL-TGR is revealed in the development of lateral line system, which is crucial in fish for food detection and predator avoidance. Most likely, RL-TGR is one of the first components in the signaling cascade; therefore, its inactivity might affect the entire signaling pathway directing the early developmental processes. Further studies are required to identify the endogenous GPCR with which RL-TGR interacts in various tissues and the downstream molecules that are stimulated during the signaling.

## Methods

### Ethics statement

This study was approved by the Institutional Animal Care and Use Committee at Georgia Institute of Technology (A14039). All animal work was performed according to procedures approved by the Institutional Animal Care and Use Committee at the Georgia Institute of Technology.

### Zebrafish strains

Adult fish and embryos (AB/Tuebingen - wildtype) were raised and maintained under standard laboratory conditions^27^. Breeding wild type strains were maintained at 28 °C on a 14 h light/10 h dark cycle. Embryos were collected by natural spawning, staged according to Kimmel and colleagues^28^, and raised at 28 °C in fish water (Instant Ocean, 0.1% methylene blue) in Petri dishes. We report the embryonic ages in hours post fertilization (hpf) and days post fertilization (dpf).

### *In situ* hybridization and immunohistochemistry

Whole-mount *in situ* hybridization (WISH), was carried out as previously described^29^ on embryos fixed overnight in 4% paraformaldehyde/phosphate buffered saline (PBS), then rinsed with PBS-Tween (0.1% Tween-20), dehydrated in 100% methanol and stored at −20 °C until processed for WISH^30^. *rltgr* antisense riboprobes were previously *in vitro* labelled with modified nucleotides (digoxigenin, Roche). The template for the antisense RNA probe was amplified from embryonic cDNA with the following primers: forward: 5′-CCAGGATCCCTTCAGAGTTTTTATGTATCTGGAC-3′, reverse: 5′-GCGGCCGCTTTCAAACAGTCTGTGATCG-3′. Immunohistochemistry on whole-mount zebrafish embryos was performed as previously described^31^ using the following antibodies: rabbit anti-RL-TGR (1:200; custom synthesized, Covance), goat anti-calretinin (1:100; Millipore Sigma), goat anti-Prox1 (1:20; R&D Systems), mouse anti- acetylated tubulin (1:1000; Sigma), mouse anti-parvalbumin (1:200; Millipore Sigma) and fluorescently conjugated Alexa antibodies (1:200; Molecular Probes). For coimmunostaining with Prox1 or calretinin or acetylated tubulin or parvalbumin, embryos were incubated with all primary antibodies and finally with secondary antibodies. Embryos were either mounted in Vectashield with DAPI (Vector Laboratories) and imaged on a Zeiss LSM 700 A or B confocal microscope or embedded in 1% agarose and imaged on a Zeiss Lightsheet Z.1 microscope. The neuromasts were assessed by staining the embryos with SYTOX green dye (Thermo Fisher Scientific) and observed under Zeiss epifluorescence microscope. SYTOX green stains the nuclei of neuromast cells organized in rosette pattern. For checking the specificity of the anti-RL-TGR antibody, HEK293 cells were grown at 37 °C with 5% CO_2_ in DMEM supplemented with 100 IU/mL of penicillin and 100 µg/mL of streptomycin and 10% fetal bovine serum. Cells were transfected with a pcDNA3.1 (+) vector containing *rltgr* as described previously^5^. Immunostaining with anti-RL-TGR antibody of the untransfected and transfected HEK293 cells was performed as described above.

### Morpholino injections

Morpholino (MO) injections were carried out on 1- to 2-cell stage embryos. To block *rltgr* mRNA translation, two non-overlapping ATG-targeting morpholinos were synthesized (Gene Tools, LLC): *rltgr* MO1, 5′- GGTTTCATCTGTTAAAACGGTCTGT-3′ and *rltgr* MO2, 5′ -ATGTCCAGATACATAAAAACTCTGA-3′. Unless otherwise indicated, 2 ng of *rltgr* MOs was injected. A standard control MO (4 ng; 5′-CCTCTTACCTCAGTTACAATTTATA-3′) targeting a human beta-globin intron mutation was used as a negative control (Gene Tools, LLC). Sense-strand-capped *rltgr* mRNA was synthesized with mMESSAGE mMACHINE kit (Ambion). For rescue experiments, embryos were injected with 200 pg *rltgr* mRNA with 2 ng *rltgr* MO1. Statistical analyses were carried using either Student’s *t* test for independent samples or two-way ANOVA followed by Tukey tests when appropriate.

### Alcian blue staining

Five dpf embryos were fixed overnight in 4% paraformaldehyde/PBS, then rinsed with PBS-Tween and dehydrated in 100% methanol at −20 °C until use. On the day of staining, embryos were washed with PBS-Tween (PBT) several times and then bleached in 30% hydrogen peroxide for 2 h. After that embryos were rinsed twice with 1 ml of PBT and transferred into an alcian blue 8GX (Alfa Aesar) solution (1% concentrated hydrochloric acid, 70% ethanol and 0.1% alcian blue) and stained overnight. Next day, embryos were rinsed three to four times with 1 ml acidic ethanol (5% concentrated hydrochloric acid, 70% ethanol, HCl-EtOH) for 20 minutes. The embryos were then rehydrated in water and stored in 1 ml of glycerol-potassium hydroxide solution for microscopic observation.

### Reverse transcription quantitative PCR (RT-qPCR)

Reverse transcriptions (RTs) were performed using 1 μg DNase I treated (PerfeCTa DNaseI, Quantabio) total RNA in presence of random hexamers (Verso cDNA synthesis kit, Thermo Scientific). Real-time PCRs were carried out in a total volume of 15 μl containing 1 × iTaq™ Universal SYBR Green Supermix (BioRad), using 1 μl of the RT reaction. PCRs were performed using the StepOne Plus qPCR system (Applied Biosystems). For normalization purposes, 18 S ribosomal RNA level was tested in parallel with the gene of interest. The following primers were used: *rltgr_*sense 5′-CTGGACATTATAGATATGGTTGAGACT-3′; *rltgr_*antisense 5′-TTTAACAATGCTCTCATGTTTCCC-3′; 18S_sense 5′-CGGAGGTTCGAAG ACGATCA-3′; 18S_antisense 5′-TCGCTAGTTGGCATCGTTTATG-3′.

### Characterization of expression patterns for *rltgr* by RNA-Seq

RNA-Seq data from 4 zebrafish tissues: adult zebrafish head (accession code SRR1028002-4, SRR1648856, SRR527834), olfactory epithelium (ERR375744-47, ERR375748-49), muscle (SRR1609753-55, SRR891510, ERR145653) and intestine (SRR1609744-46, SRR 1524245, SRR1562529) were downloaded from Sequence Read Archive (SRA), NCBI. Briefly, we mapped all reads per time-point independently back to the zebrafish genome (GRCz10; Ensembl) with CLCGenomics Workbench (version 8.5.1) and reads counts mapped to *rltgr* gene were recorded. We also mapped the reads from heads and intestines to both genic and intergenic regions. For comparison, the expression level of *rltgr* in a tissue was defined by the number of uniquely mapped reads in *rltgr* divided by one thousandth of the whole exon length of *rltgr*, and then was normalized by dividing by one millionth of the total number of valid reads in the respective samples.

## Electronic supplementary material

Supplementary Information
