# Supplementary material for: Zebrafish aversive taste co-receptor is expressed in both chemo- and mechanosensory cells and plays a role in lateral line development
Source: Sci Rep. 2017 Oct 18;7:13475. doi: 10.1038/s41598-017-14042-3 (PMC5647393; doi:10.1038/s41598-017-14042-3)
Supplement: Supplementary file 1 — Supplementary Information [file 41598_2017_14042_MOESM1_ESM.pdf]

Supplementary information

Zebrafish aversive taste co-receptor is expressed in both chemo- and mechanosensory cells and plays a role in lateral line development

Nazia Mojib, Jin Xu, Zinka Bartolek, Barry Imhoff, Nael A. McCarty, Chong Hyun Shin, and Julia Kubanek

Supplementary Figure S1.

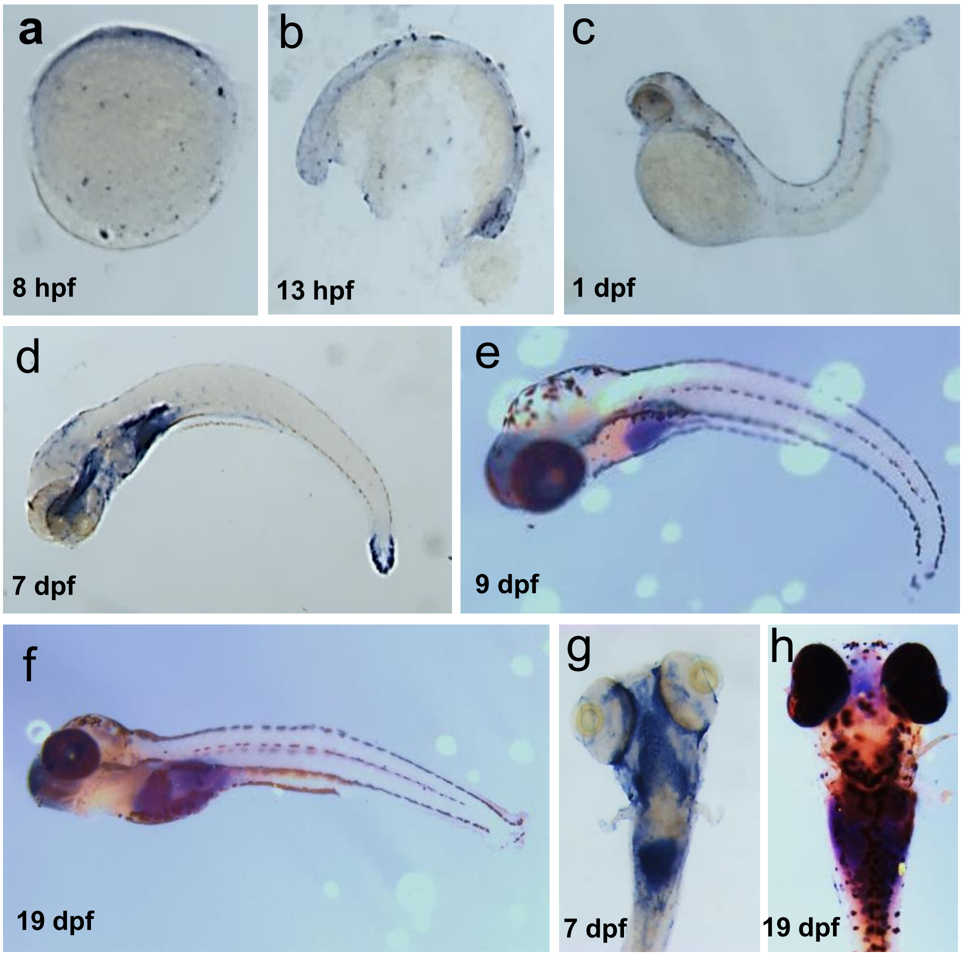

Figure S1. Spatiotemporal expression of zebrafish *rltgr* mRNA*.* Wild type embryos were stained by ISH to identify the cells or tissues that express *rltgr* (a-h). (a-f) are whole mount embryos is lateral view, anterior to the left. (g-h) is a dorsal view.

Supplementary Figure S2.

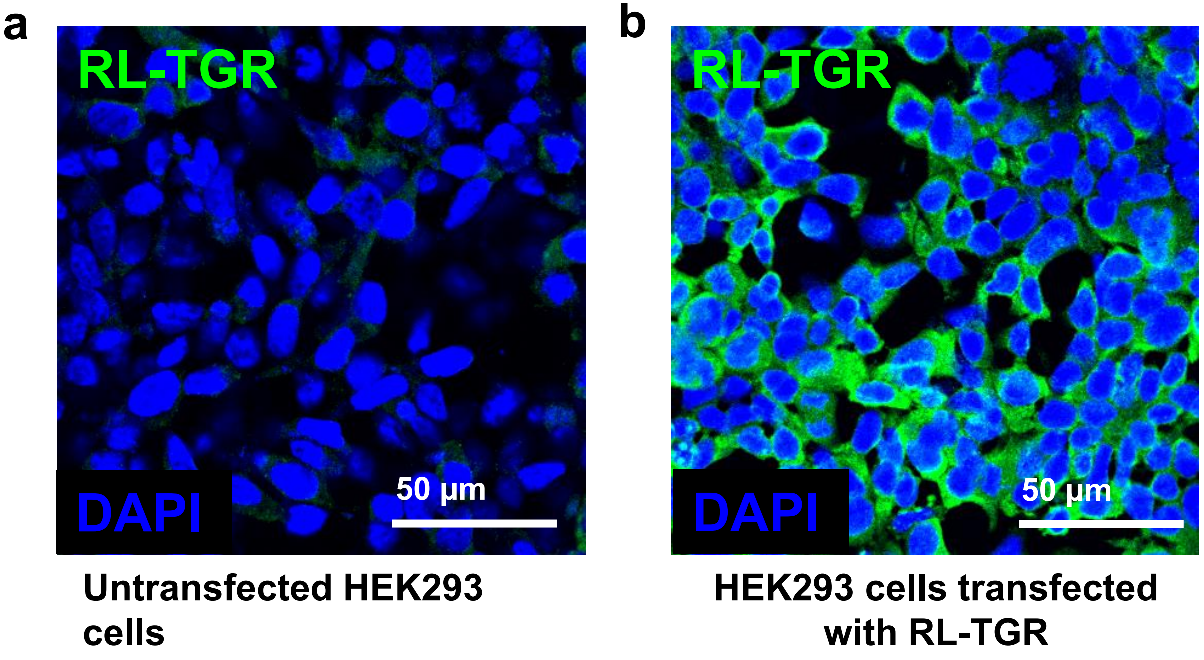

Figure S2. Specificity of custom-generated anti-RLTGR antibody with RL-TGR protein. (a) Confocal image of the untransfected Human Embryonic Kidney (HEK) 293 cells immunostained with anti-RL-TGR antibody (green) followed by staining with DAPI that stains nuclei blue. (b) Confocal image of HEK293 cells transfected with mammalian expression vector, pcDNA3.1 (+) containing coding region of *rltgr* and then immunostained with anti-RL-TGR antibody (green) followed by staining with DAPI (blue). The green fluorescence observed in cells expressing recombinant RL-TGR protein indicates that the custom generated antibodies recognize specifically the RL-TGR protein.

Supplementary Figure S3.

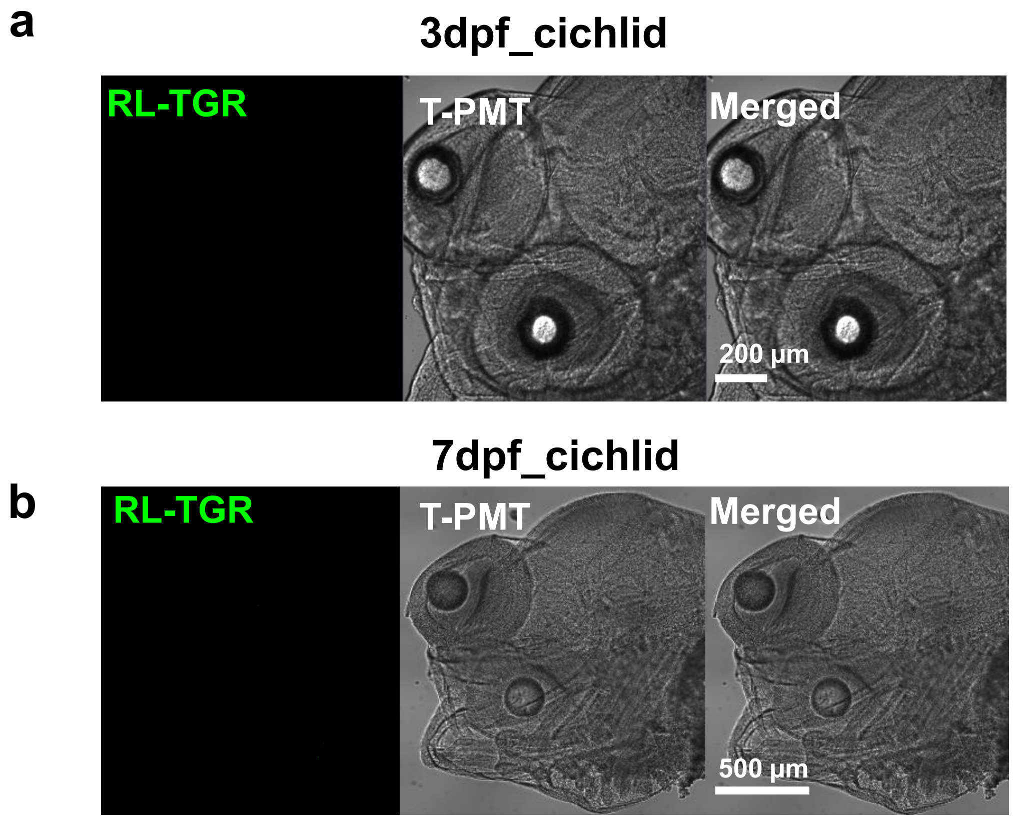

Figure S3. Negative staining with anti-RL-TGR antibody in cichlid embryos (*Maylandia zebra*) that do not have *rltgr* gene. (a) Confocal image showing 3 dpf wild type cichlid embryos immunostained with anti-RL-TGR antibody (green). (b) Confocal image showing 7 dpf wild type cichlid embryos immunostained with anti-RL-TGR antibody (green). There was no green fluorescence detected in both stages in cichlid because cichlid genome do not contain *rltgr* gene and therefore do not have RL-TGR protein. This negative control further validates the specificity of anti-RL-TGR antibody in reacting with RL-TGR protein.

Supplementary Figure S4.

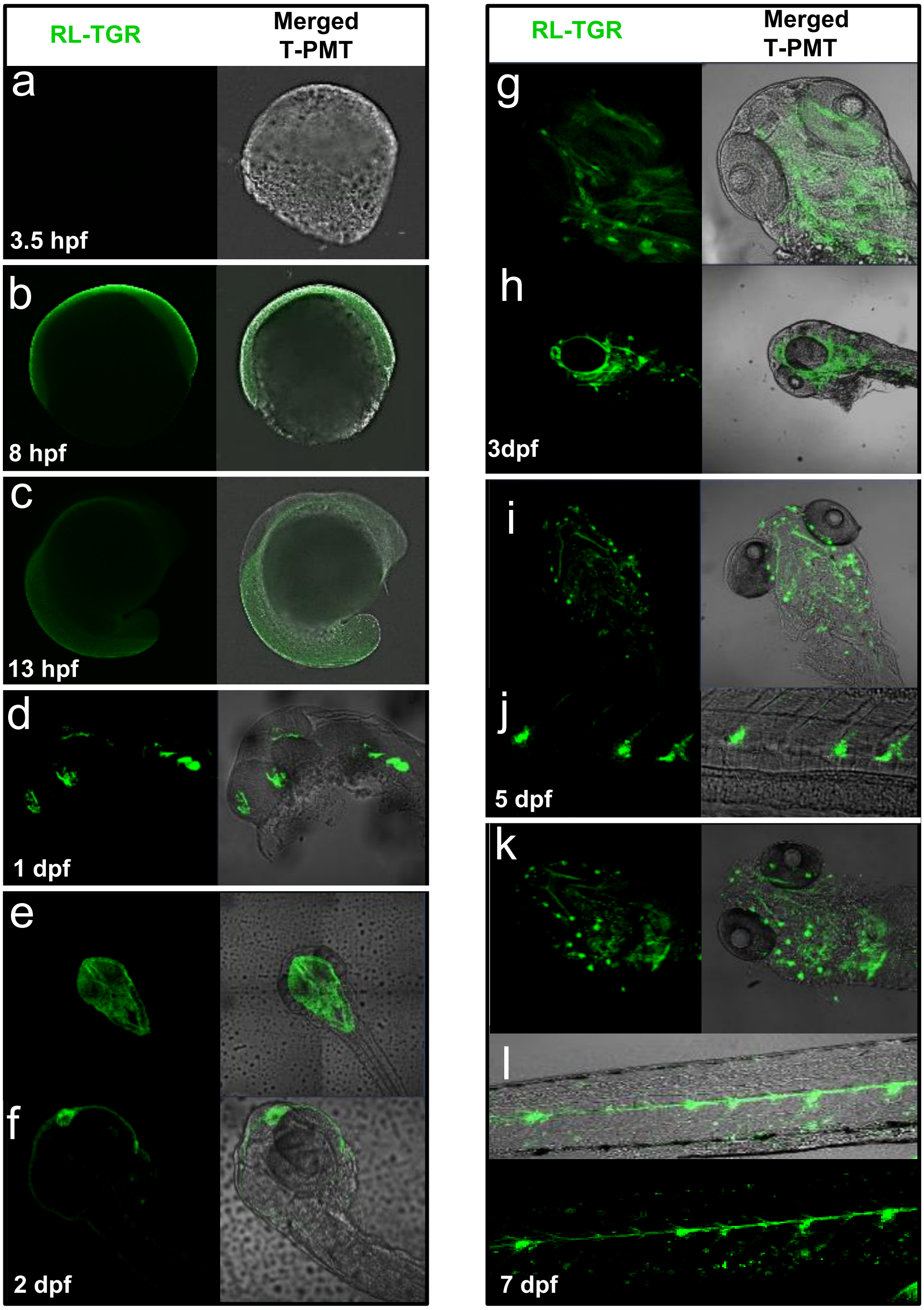

Figure S4. Spatiotemporal expression of zebrafish RL-TGR protein. Wild type embryos were immunostained with anti RL-TGR antibodies to identify the cells or tissues that express RL-TGR protein (a-l). (a-d,f,h,j,l) are whole mount embryos in lateral view, anterior to the left. (e,g,i, k) is a dorsal view.

Supplementary Figure S5.

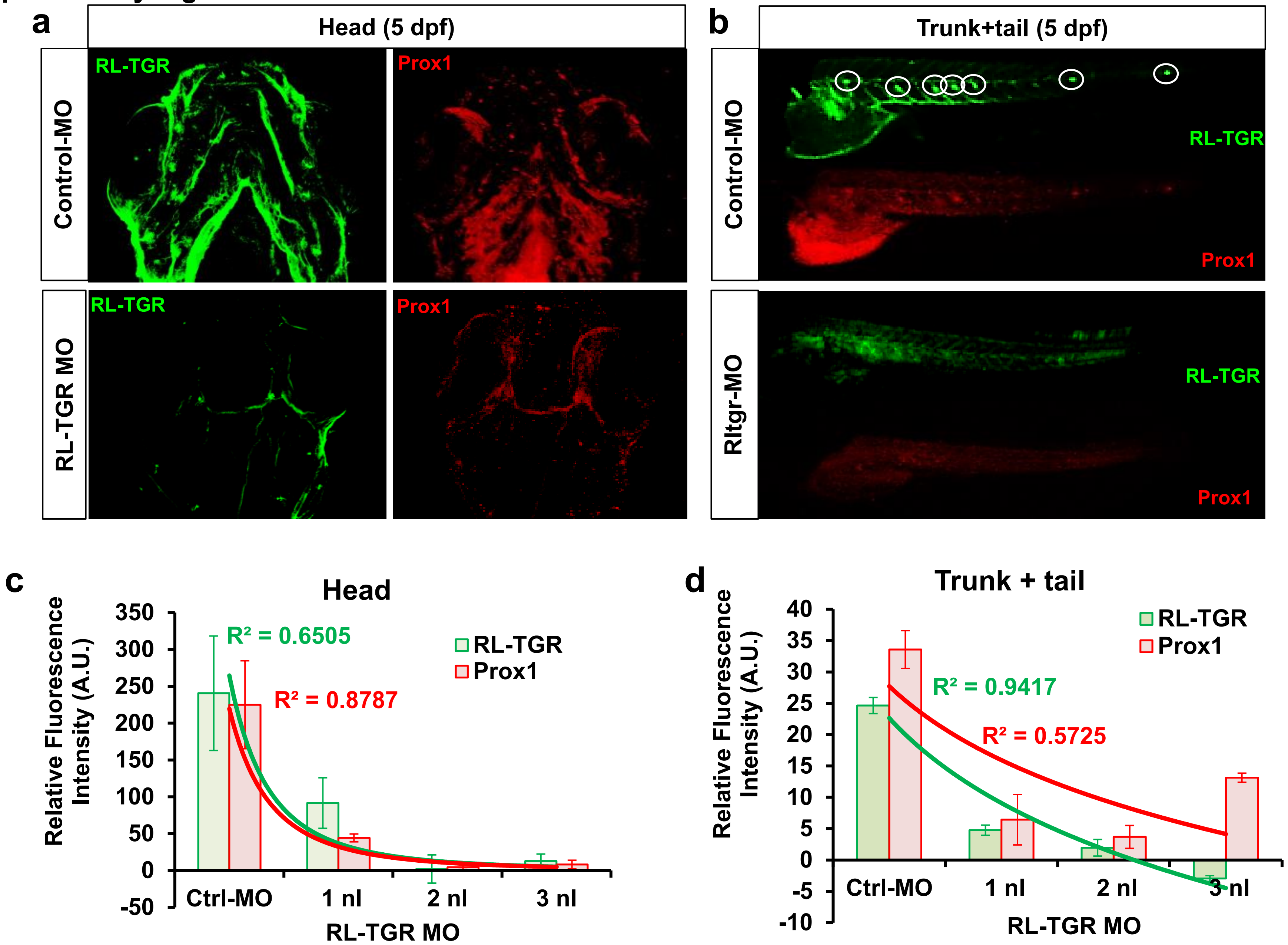

Figure S5. (a) Double immunostaining of RL-TGR and Prox1 in embryos injected with control and RL-TGR MO (5dpf). (b) Relative fluorescence intensity of RL-TGR and Prox1 in embryos injected with control and RL-TGR MO (5dpf).

Supplementary Figure S6.

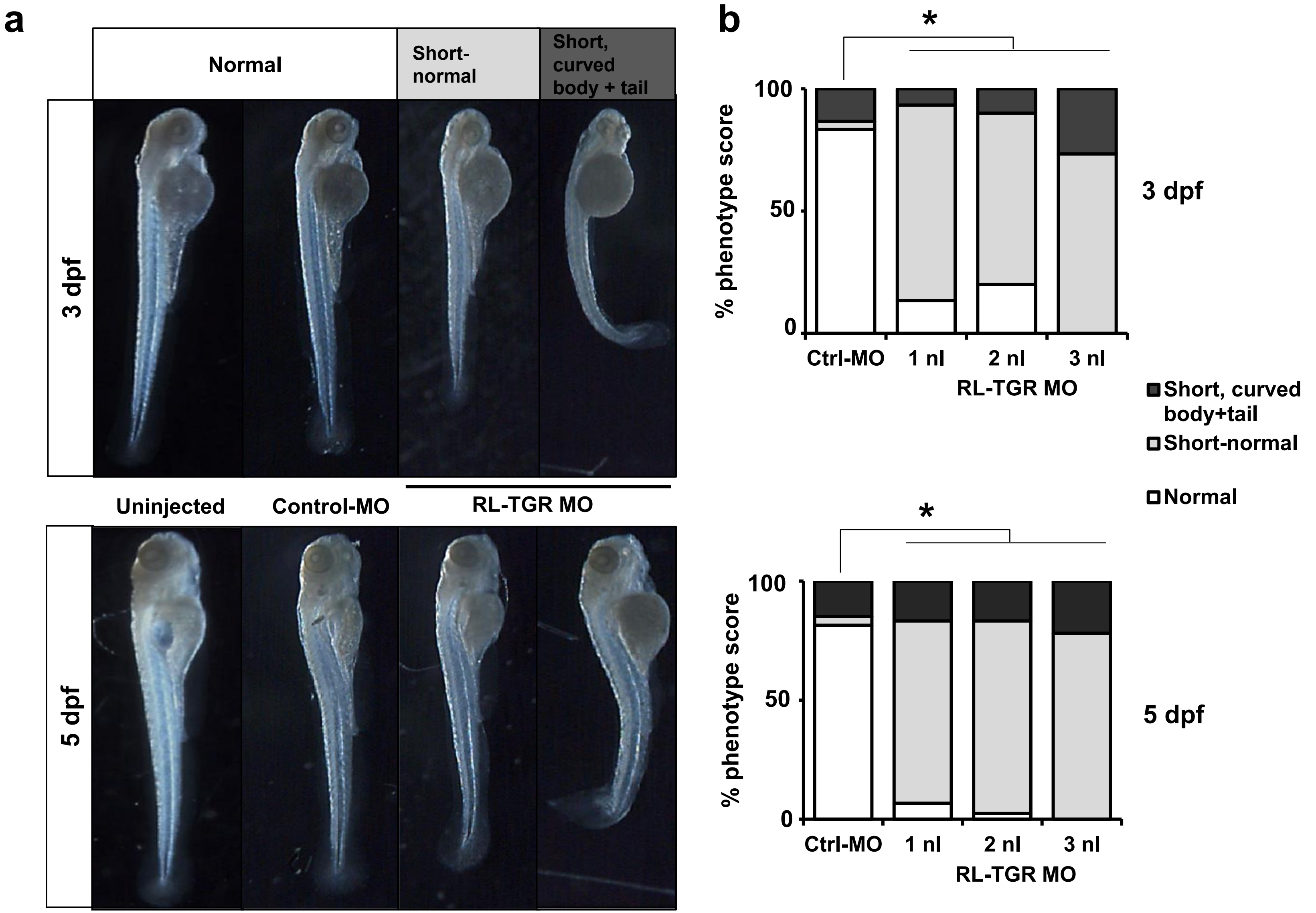

Figure S6. Dose dependent effect of RL-TGR morpholino. (a) Assessment of morphological defects in the morpholino injected embryos compared to control, at 3 and 5 dpf (b) Percent phenotype scores of RL-TGR morphants and control morphants (*P<0.0001, n=30-81).

Supplementary Figure S7.

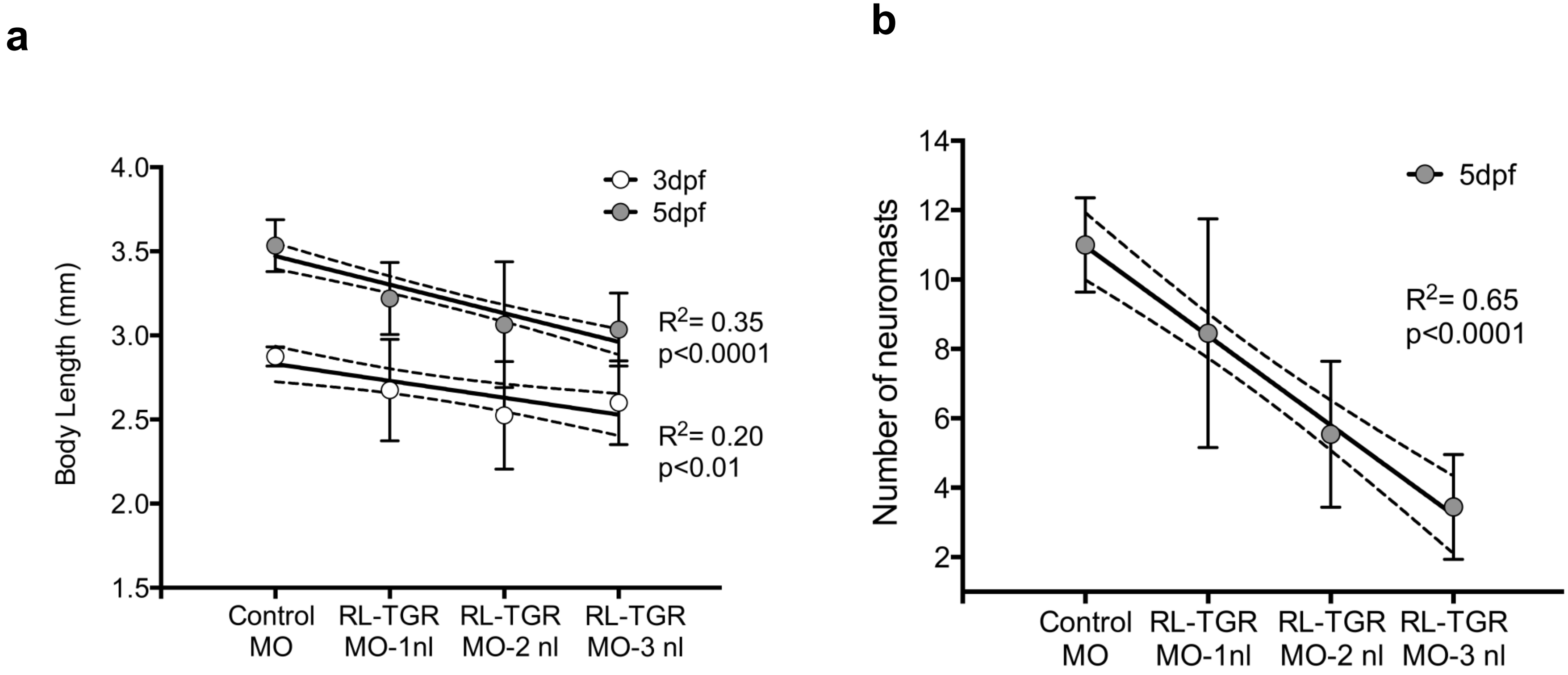

Figure S7. Dose dependent effect of RL-TGR morpholino on body lengths and number of neuromasts. (a) The body length of the injected populations both at 3 and 5 dpf is shown as a bar graph which shows significant dose dependent decrease in the body length, compared to control (n=150). (b) The number of neuromasts of the injected populations at 5 dpf is shown as a bar graph which indicates significant dose dependent decrease in the number of neuromasts, compared to control (n=59).
